# Capillary-Driven Flow in Triangular Nanofluidic Channel Fabricated via Nanoimprint Lithography with Heterogeneous Semiconductor Materials

**DOI:** 10.1021/acsomega.6c07925

**Published:** 2026-09-15

**Authors:** Yi-Hsin Wang, Kang-Yuan Lee, Chung-Hsiang Lin, Chao-Min Cheng

**Affiliations:** † Institute of Biomedical Engineering, 34881 National Tsing Hua University, Hsinchu 300044, Taiwan; ‡ Quantum NIL Corporation, Miaoli 350401, Taiwan

## Abstract

In this study, hollow single- and triple-layer triangular nanofluidic channels with heterogeneous semiconductor walls composed of gallium nitride (GaN) and silicon nitride (Si_3_N_4_) were fabricated via nanoimprint lithography and PECVD-based thin-film deposition to investigate capillary-driven flow behaviors of liquids commonly used in biological applications. The fabricated single-layer channels enabled quantitative characterization of capillary-driven flow transport, while the triple-layer channels demonstrated the feasibility of extending the fabrication strategy to multilayer nanofluidic architectures. The measured flow velocities inside single-layer nanofluidic channels for the test liquids ranged from 0.17 to 16.86 μm/s. Based on the experimentally measured flow velocities, the calculated capillary pressures, pressure drops, and average wall shear stresses were 0.46–1.27 MPa, 27–113 Pa, and 1.32–3.65 kPa, respectively. The results demonstrated that liquid viscosity and surface wettability would influence capillary-driven flow transport, providing quantitative insights into passive capillary flow in nanofluidic channels and establishing a foundation for the design of future biomedical and analytical nanofluidic devices.

## Graphical abstract

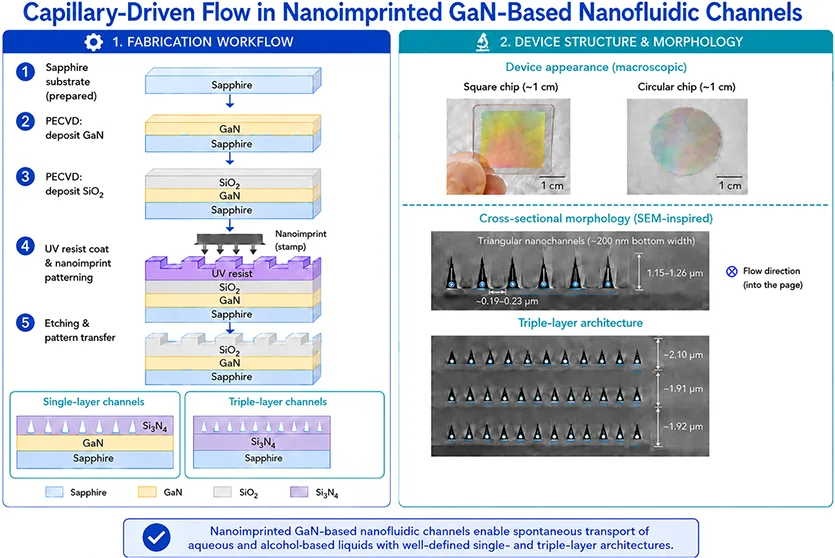

## 1 Introduction

Capillary-driven flow has emerged as an effective strategy for passive liquid transport in micro- and nanofluidic systems, eliminating the need for external pumping while maintaining simple device architectures and low energy consumption. These advantages make capillary transport particularly attractive for biomedical and bioanalytical applications, such as point-of-care diagnostics, biosensing, molecular separation, and single-cell analysis, where precise handling of minute liquid volumes is required. At the nanoscale, capillary pressure becomes increasingly dominant and facilitates spontaneous sample loading, reagent delivery, and analyte confinement within nanofluidic devices. Consequently, capillary-driven flow transport has become a fundamental mechanism in a wide range of applications, including DNA manipulation, protein analysis, and extracellular vesicle isolation.^1–3^

However, as characteristic dimensions approach the nanoscale, deviations from classical capillary theory, originally developed for macroscopic and microfluidic systems, become increasingly pronounced. These deviations arise from factors such as material heterogeneity, enhanced viscous dissipation, and the amplified influence of liquid-solid interfacial interactions, all of which are known to critically affect transport behaviors in nanoconfined environments.^4,5^ Extensive analytical and experimental studies have been conducted on capillary-driven flow in circular and rectangular micro- and nanofluidic channels, providing insights into flow behaviors.^6,7^ By contrast, triangular nanofluidic channels, despite their frequent emergence in advanced nanofabrication processes, remain comparatively underexplored. Such geometries naturally arise from anisotropic etching, corner lithography, and multilayer deposition techniques commonly employed in semiconductor manufacturing. From a nanofluidic perspective, the triangular cross section introduces sharp corners and spatially varying hydraulic resistance,^8^ which can substantially alter the velocity profile, shear stress distribution, and overall flow behaviors. Moreover, state-of-the-art semiconductor-based nanofluidic platforms increasingly integrate heterogeneous materials such as gallium nitride (GaN) and silicon nitride (Si_3_N_4_) to achieve multifunctionality, chemical robustness, and compatibility with electronic or optoelectronic components. These materials exhibit markedly different surface energies and wettability characteristics, leading to non-uniform boundary conditions along the channel perimeter.^9^ In biomedical nanofluidic platforms, such wettability heterogeneity could influence protein adsorption and biomolecular transport, thereby directly impacting device performance and data interpretation.

Nanoimprint lithography in this study was employed to fabricate heterogeneous semiconductor nanofluidic channels for biomedical-related applications, extending its use beyond conventional semiconductor manufacturing. Nanoimprint lithography is a versatile pattern-transfer technique capable of producing nanoscale structures with high resolution, low cost, and high throughput.^10^ Compared with conventional nanofabrication approaches, nanoimprint lithography offers several advantages, including simplified processing, scalability, and the ability to generate complex three-dimensional nanostructures.^11^ These characteristics make nanoimprint lithography particularly attractive for the fabrication of nanofluidic devices requiring precise geometric control and multilayer integration. By combining GaN and Si_3_N_4_ within a single nanoscale architecture, nanoimprint lithography provides an effective platform for investigating capillary-driven flow transport in heterogeneous semiconductor nanofluidic systems. Heterogeneous GaN and Si_3_N_4_ nanofluidic channels with isosceles triangular cross sections were fabricated to investigate capillary-driven flow transport. By integrating nanoimprint lithography with conformal thin-film deposition, single-layer and triple-layer channel architectures were fabricated and experimentally characterized. The ability of these structures to support capillary-driven flow transport was further investigated through flow characterization. The successful realization of functional multilayer nanofluidic channels demonstrated the feasibility and capability of the proposed fabrication strategy. Using these nanoimprint-lithography-fabricated channels, average flow velocity, volumetric flow rate, and contact angles on GaN and Si_3_N_4_ were quantified. Combining these experimental measurements with analytical fluidic mechanics enabled the effects of channel geometry and heterogeneous surface wettability on capillary-driven flow transport under nanoconfined conditions to be elucidated. Such studies remain relatively scarce for heterogeneous triangular nanofluidic channels, despite their increasing relevance in semiconductor-based nanofluidic devices. The results presented help establish a framework for understanding and optimizing capillary-driven flow transport in multifunctional semiconductor nanofluidic platforms.

## 2 Methods and Materials

### 2.1 Nanofluidic Channel Fabrication

The rationale for selecting GaN and Si_3_N_4_ is twofold. First, both materials are optically transparent, allowing direct observation of capillary-driven flow transport inside the nanofluidic channels using a conventional optical microscope. This greatly facilitates visualization and quantitative characterization of the flow transport process. Second, GaN and Si_3_N_4_ are well-established semiconductor materials that are widely employed in semiconductor manufacturing and are compatible with mature thin-film deposition and nanofabrication technologies. Since the objective of this work is to investigate capillary-driven flow transport in heterogeneous semiconductor nanofluidic channels, these materials provide a representative and practical material platform for demonstrating the proposed fabrication strategy. Single- and triple-layer nanofluidic channels were fabricated via nanoimprint lithography. The single-layer nanofluidic channel fabrication process is illustrated in Figure 1(a) and the detailed steps are described as follows.

(1) Prepare the sapphire substrate.
(2) A GaN epitaxial layer was grown using plasma-enhanced chemical vapor deposition (PECVD). The growth was carried out at a chamber temperature of 1145 °C on a sapphire substrate and using nitrogen as the carrier gas. Trimethylgallium (TMGa) and ammonia (NH_3_) were introduced as precursors to facilitate gas-phase and surface chemical reactions within the reactor. Through high-temperature decomposition, Ga and N atoms were generated and subsequently combined to form a solid GaN thin film with a thickness of approximately 5 μm on the sapphire substrate.
(3) A silicon dioxide (SiO_2_) thin film was then deposited via PECVD. The precursors were vaporized and activated in low-temperature plasma at 300 °C. Plasma cleaning was performed prior to deposition to ensure a clean surface. Vaporized SiH_4_/NH_3_/N_2_O gases reacted on the substrate surface, forming a uniform SiO_2_ thin film.
(4) A layer of UV resist was spin-coated onto the SiO_2_ surface. During spin coating, centrifugal force and fluid dynamics caused the resist to spread uniformly, while excess solution was expelled from the edges, accompanied by solvent evaporation, resulting in a thin and uniform resist layer.
(5) Nanoimprint lithography was applied to transfer the pattern onto the photoresist layer, forming periodic gratings with a pitch of 977 nm. The pattern structure of the mold was designed by CAD software, and then the mold was fabricated by precisely controlled high-resolution electron beam writing.
(6) The grating patterns were subsequently transferred into the GaN layer by inductively coupled plasma (ICP) dry etching, with an etching depth of approximately 1 μm. This step enabled the formation of high-aspect-ratio structures with vertical sidewalls. An aspect ratio (height-to-width) greater than 1 was required to ensure the feasibility of subsequent thin-film deposition.
(7) After etching, a Si_3_N_4_ thin film was deposited using PECVD to seal the surface and complete the formation of the nanofluidic channels.

**Figure 1:**
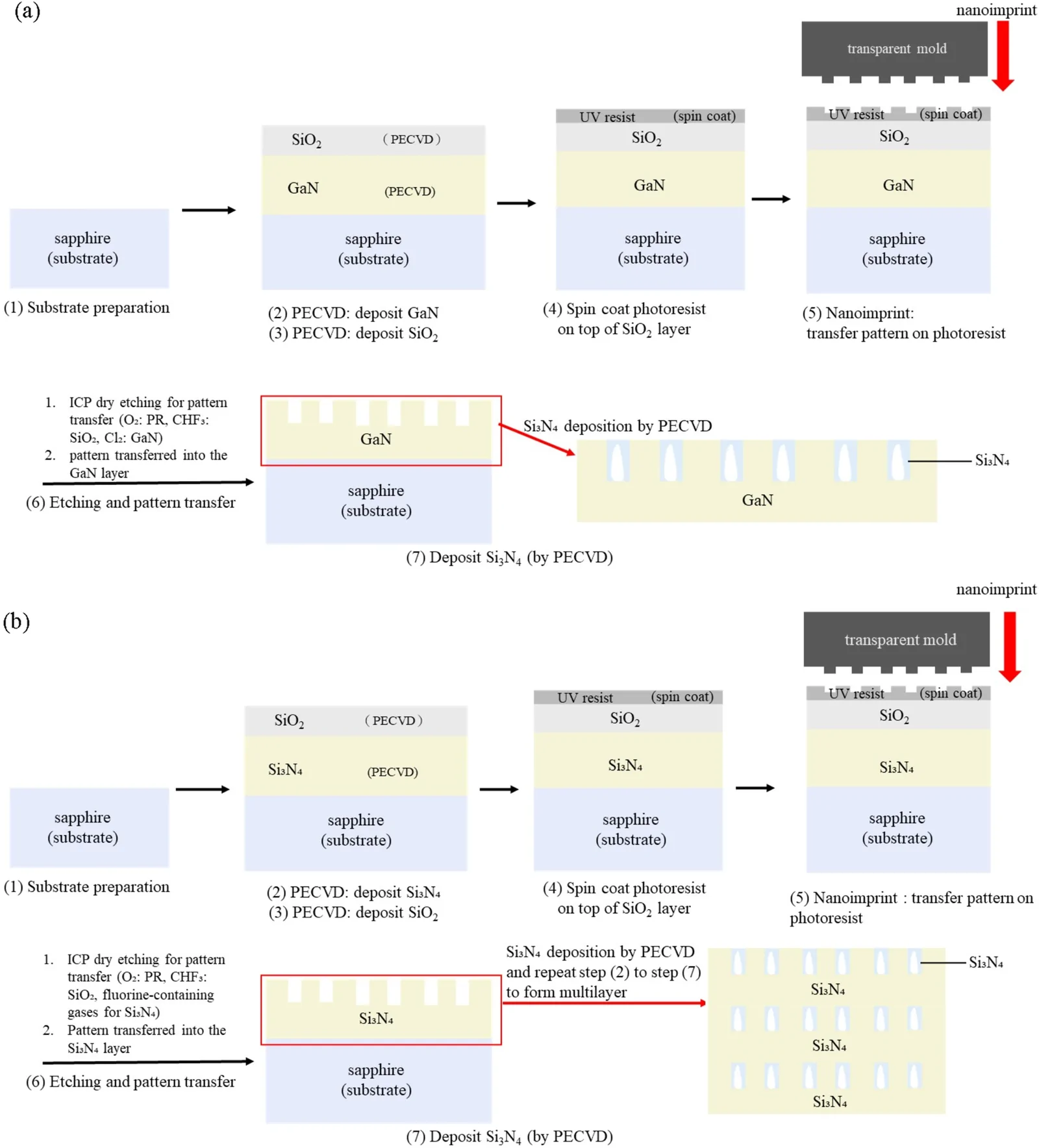
(a) Fabrication of single-layer nanofluidic channels and (b) triple-layer nanofluidic channels. The difference between single-layer nanofluidic channels and triple-layer nanofluidic channels was the deposition on prepared sapphire substrate of a different kind of material via PECVD. For single-layer nanofluidic channel, GaN was deposited, whereas Si_3_N_4_ was deposited for triple-layer nanofluidic channel. Triple-layer nanofluidic channel repeat step (2) to step (7) to form multiple layer structure.

The three-layer nanofluidic channel fabrication process followed the same procedure as that for the single-layer channels, except that the intermediate GaN layer was replaced with Si_3_N_4_. To construct the three-layer nanofluidic channels, steps 2-7 were repeated. Because the deposited Si_3_N_4_ layer exceeded the desired thickness, it was subsequently thinned by inductively coupled plasma (ICP) dry etching. A schematic illustration of this fabrication process is shown in Figure 1(b).

### 2.2 Experimental Procedure for Observing Capillary-Driven Flow in Nanofluidic Channels

The experimental setup and validation procedure are illustrated in Supporting information (Figure S1). The single-layer nanofluidic channel sample (Quantum NIL Corporation, Miaoli, Taiwan) was positioned with the imprinted nanofluidic channels facing upward. The sample was then sectioned along a direction perpendicular to the nanofluidic channel orientation to expose the cross-sectional structure of the nanofluidic channels. After exposure, the test liquid (100 μL of test liquid per trial) was dispensed onto the cross-section. The test liquids included pure methanol (Cat. No. 3016-68, Macron Fine Chemicals, USA), pure ethanol (Cat. No. E/0650DF/15, Fisher Chemical, U.K.), methanol–ethanol mixtures, 1× phosphate buffered saline (Cat. No. P4417-100TAB, Sigma-Aldrich, USA), and 1 mM ethylenediaminetetraacetic acid solutions (Cat. No. 8991-01, J.T.Baker, USA). Red ink (INK-30-R, PILOT, Japan) was added solely to improve the visualization of the liquid front during video recording. Without the red ink, the liquid front could not be clearly distinguished, making it difficult to accurately determine the propagation distance and flow velocity. To minimize the potential influence on the experimental results, the red ink was added at a tiny concentration of 1/50 by volume, which was the minimum amount required to provide sufficient optical contrast for image analysis. Videos of the flow behavior inside the nanofluidic channels (Supporting Information, Videos S1 and S2) were recorded using an iPhone 15 Pro smartphone (Apple Inc., USA) mounted on the eyepiece of an optical microscope (Model MX50, Olympus Corp., Japan).

The actual flow distance of the test liquid was estimated using the following procedure. After the liquid flow process was recorded, the length of the video image from top to bottom was measured using a ruler. Assuming that the measured image length was *x* cm and that a total of *y* nanofluidic channels were identified in the recorded video, the corresponding actual length was determined based on the average channel width of 0.2 μm. Therefore, the total actual width represented in the image was 0.2*y* μm, indicating that a ruler-measured *x* cm in the image corresponded to 0.2*y* μm in the nanofluidic channels. Based on this calibration relationship, if the displacement of the liquid front in a video segment was measured as *z* cm and the corresponding elapsed time was *t* seconds, the average velocity was calculated using the following equation

1 $$average\;velocity=\frac{0.2y\times \frac{z}{x}}{t}\mu m/s$$

An uncertainty analysis was conducted for the velocity measurements obtained from smartphone videos. The video was recorded at 24 frames per second, corresponding to a frame interval of 0.0417 s. Considering single-frame uncertainty in identifying both initial and final positions of the liquid front, the combined temporal uncertainty was estimated to be 0.059 s. The liquid-front displacement and image length were measured using a ruler with a minimum division of 1 mm, corresponding to a combined spatial uncertainty of ±0.071 cm (Supporting Information).

## 3 Results and Discussion

### 3.1 Single- and Triple-Layer Nanofluidic Channels

Representative images of the fabricated nanofluidic channels are shown in Figure 2. Macroscopic photographs were acquired using a smartphone camera, while the cross-sectional morphologies were characterized via scanning electron microscopy (JSM-IT800, JEOL Ltd., Japan). The characteristic dimensions of the fabricated nanofluidic channels, including the cross-sectional area and hydraulic diameter, were all within the nanometer scale. By repeating the fabrication process, a triple-layer nanofluidic channel architecture was successfully fabricated. Scanning electron microscopy images confirmed the successful formation of stacked nanofluidic channel structures while maintaining well-defined channel geometries. The different geometries shown for the single-layer and triple-layer nanofluidic channels were intentionally retained because they represented the actual morphologies obtained after fabrication. Although the overall fabrication procedure was essentially identical for both channel architectures, the deposited materials used in the PECVD process were different. Since different materials exhibited different deposition characteristics, including vertical and lateral deposition rates, the final cross-sectional morphology of the nanofluidic channels was consequently altered.

**Figure 2:**
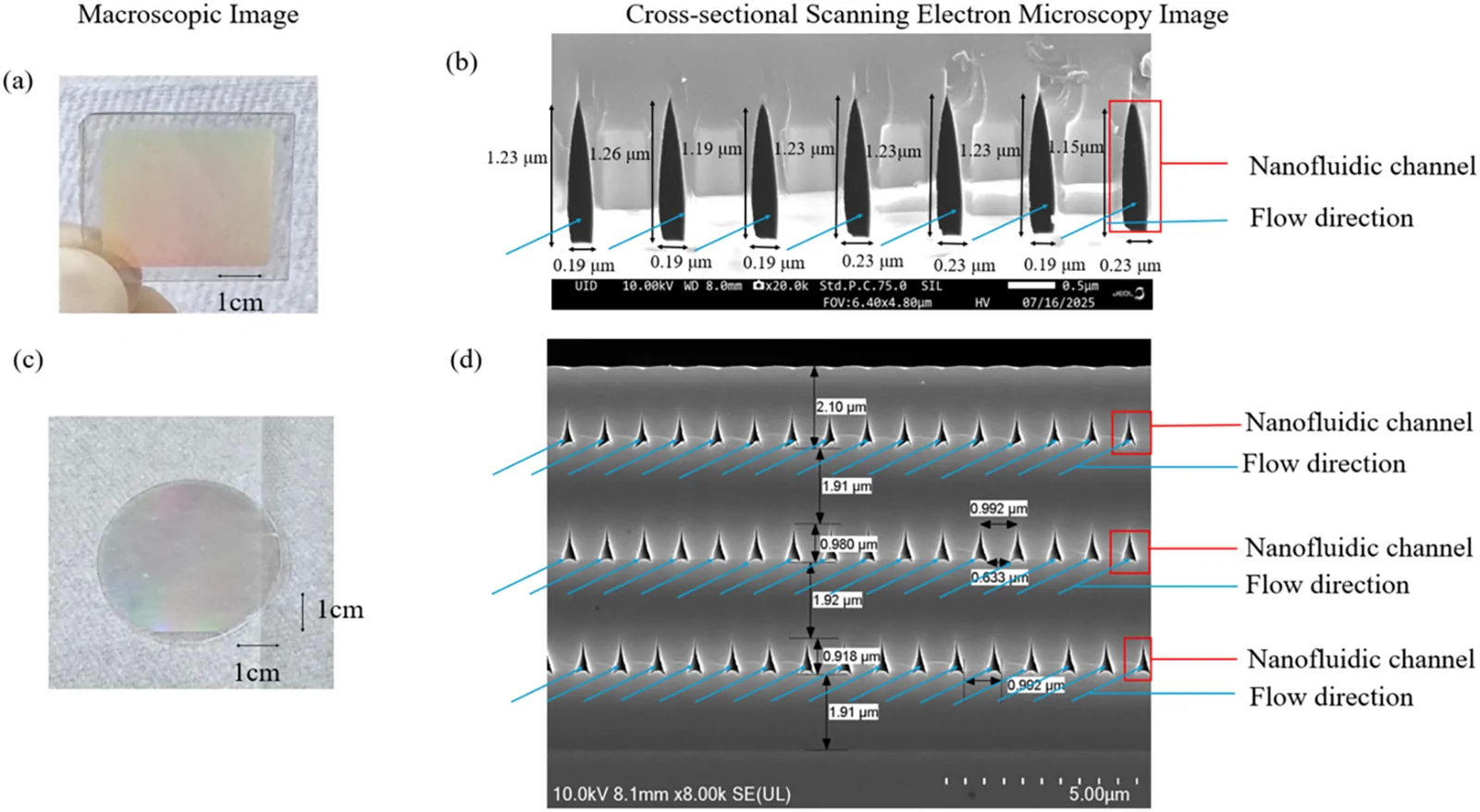
(a) Macroscopic image of single-layer nanofluidic channels, (b) scanning electron microscopy image of single-layer nanofluidic channels, (c) macroscopic image of triple-layer nanofluidic channels and (d) scanning electron microscopy image of triple-layer nanofluidic channels.

The fabrication of multilayer nanostructures remains challenging because it requires precise nanoscale alignment, uniform residual layer control, and minimization of fabrication defects. The successful realization of the triple-layer architecture therefore represents an important advancement in the proposed fabrication strategy.^11^ Previous studies have demonstrated multilayer nanoimprint fabrication by employing materials with different glass transition temperatures to prevent deformation of previously patterned layers during subsequent imprinting processes.^12^ By contrast, the multilayer structure demonstrated in this study was achieved through repeated deposition and pattern-transfer processes while preserving the integrity of the underlying nanofluidic channel architecture. The main objective of fabricating the triple-layer nanofluidic channels in this study was to demonstrate the successful fabrication of multilayer nanofluidic channels and to verify that they were capable of supporting capillary-driven flow transport (Supporting Information, Video S3).

### 3.2 Average Flow Velocity and Volumetric Flow Rate of Single-Layer Nanofluidic Channels

The experimentally measured flow velocities of individual nanofluidic channels ranged from approximately 0.17 to 16.86 μm/s. The mean flow velocity for each test liquid, calculated from the individual channels analyzed, ranged from 3.76 to 11.54 μm/s, as summarized in Table 1. Under the present experimental conditions, pure ethanol was unable to flow through the fabricated single-layer nanofluidic channels, and therefore no measurable liquid propagation was observed. As a result, the average flow velocity and volumetric flow rate could not be experimentally determined and were not reported in Table 1.

**Table 1:** Measured Average Velocity and Volumetric Flow Rate of Different Test Liquids Flowed inside Single-Layer Nanofluidic Channel (*N*: Number of Videos Used for Quantitative Analysis, *n*: Number of Individual Nanofluidic Channels Analyzed from Those Videos)^a^

test liquid	*N*	*n*	average velocity (μm/s)	volumetric flow rate (μm^3^/s)
100% methanol	3	13	7.34 ± 4.29	0.95 ± 0.56
80% methanol + 20% ethanol	3	13	3.80 ± 1.85	0.49 ± 0.24
60% methanol + 40% ethanol	3	36	11.54 ± 4.21	1.50 ± 0.55
40% methanol + 60% ethanol	3	12	7.21 ± 3.33	0.94 ± 0.43
1× phosphate buffered saline	3	9	3.76 ± 2.87	0.49 ± 0.37
1 mM ethylenediaminetetraacetic acid	3	19	10.82 ± 5.82	1.41 ± 0.76

a Note: Pure ethanol was not able to propagate through the fabricated single-layer nanofluidic channels under the present experimental conditions so that its average velocity and volumetric flow rate could not be experimentally determined and are not included in this table.

We extracted the meniscus position as a function of time from the recorded smartphone videos for all liquids exhibiting the measurable capillary-driven propagation. The position of the advancing liquid meniscus was tracked at different time points from the recorded smartphone videos to obtain the meniscus displacement as a function of time (X-t) for each liquid. It is noted that pure ethanol was excluded from the analysis because no measurable liquid propagation was observed under the present experimental conditions. To further examine whether the capillary filling behavior follows the classical Lucas-Washburn relationship, the corresponding X^2^-t plot was constructed and fitted using linear regression (Figure 3).

**Figure 3:**
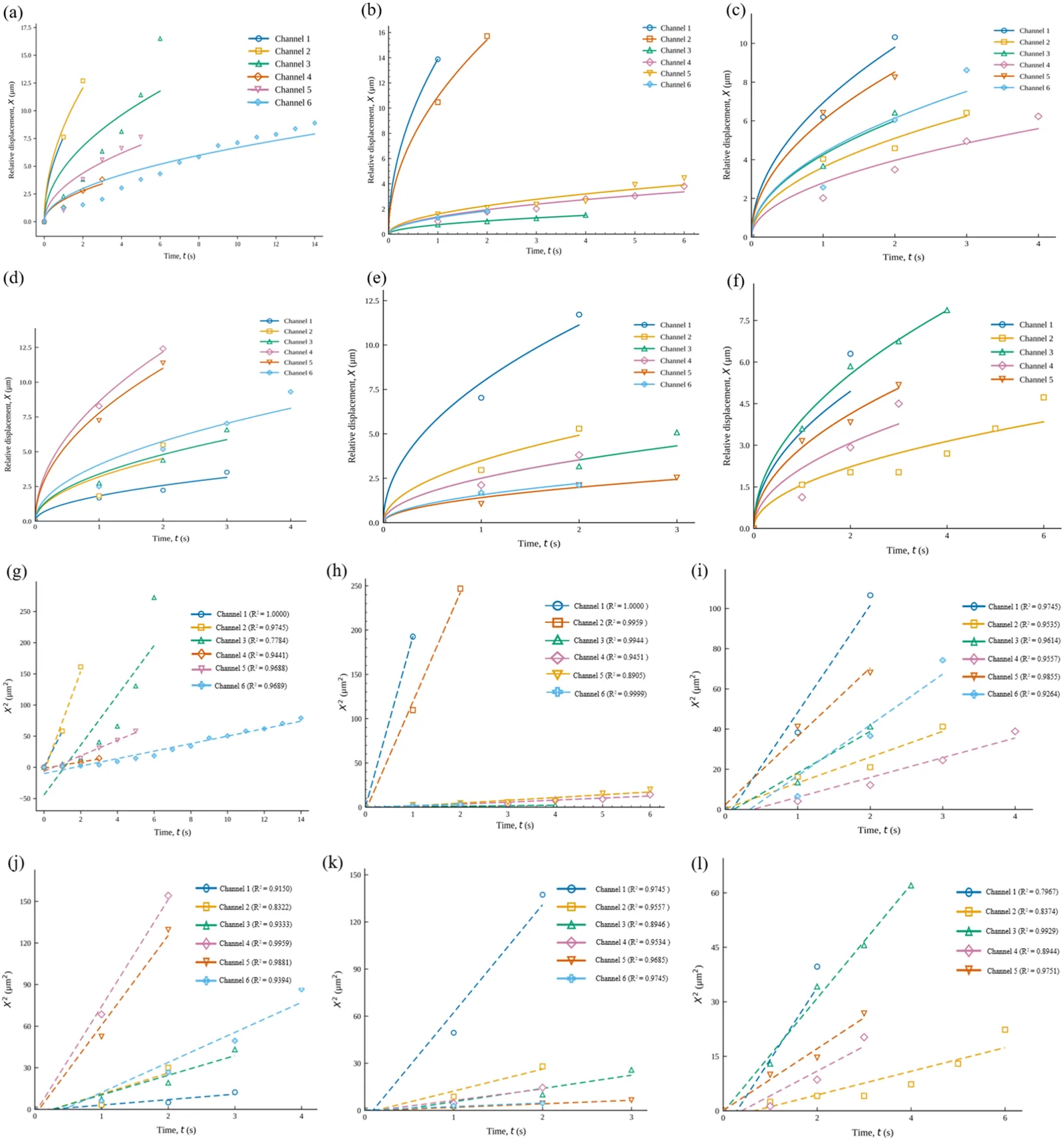
Relative displacement as a function of time of (a) 1× phosphate buffered saline, (b) 1 mM ethylenediaminetetraacetic acid, (c) 100% methanol, (d) 80% methanol + 20% ethanol, (e) 60% methanol + 40% ethanol, (f) 40% methanol + 60% ethanol and Lucas-Washburn analysis of squared meniscus displacement versus time of (g) 1× phosphate buffered saline, (h) 1 mM ethylenediaminetetraacetic acid, (i) 100% methanol, (j) 80% methanol + 20% ethanol, (k) 60% methanol + 40% ethanol, (l) 40% methanol + 60% ethanol.

The results show that the experimentally measured meniscus displacement generally follows the characteristic *X* ∝ √*t* behavior, while the *X* ^2^ versus *t* plots exhibit approximately linear relationships with relatively high coefficients of determination (*R* ^2^), supporting the applicability of the Lucas-Washburn model under the present experimental conditions. Because the capillary filling velocity naturally decreases as the meniscus advances, the average velocity reported in this study should be regarded as an average transport parameter over the measured propagation distance rather than an instantaneous velocity. Non-monotonic variation in flow velocity is attributed to the combined influence of surface tension, dynamic viscosity, and contact angle, all of which vary simultaneously with the methanol–ethanol composition. Since the capillary pressure is proportional to the surface tension, the competition between these parameters results in a nonlinear dependence of the capillary filling velocity on liquid composition.

Comparison with previously reported capillary-driven flow studies is presented in Table 2. As shown in Table 1, the measured flow velocity did not exhibit a monotonic dependence on the methanol–ethanol composition. Instead, a nonlinear relationship between liquid composition and flow velocity was observed, suggesting that fluid transport behavior cannot be explained solely by bulk fluid properties such as viscosity. Similar nonlinear filling behavior has previously been reported for water-alcohol mixtures in nanofluidic channels.^13^ In addition, Mozaffari *et al*.^14^ investigated capillary-driven flow of water, pure methanol, pure ethanol, and highly viscous bitumen-based mixtures in fluidic channels, and reported a rapid decrease in flow velocity following the initial filling stage. This behavior is consistent with the experimental observations and the recorded videos provided in Supporting Information (Videos S1 and S2). The nanofluidic channels investigated in this study were fabricated using heterogeneous semiconductor materials (GaN and Si_3_N_4_), whereas those reported in previous studies were mainly fabricated using conventional materials such as glass or SiO_*x*_.^13–15^ Different surface energies and wettability characteristics of these materials would influence the capillary pressure and the liquid-solid interfacial interactions, thereby affecting the flow velocity.^16^ In addition, fabrication-induced nanoscale surface roughness may alter liquid-solid interfacial interactions and capillary behavior,^17^ potentially contributing to the observed differences in flow transport. Because hydraulic resistance increases rapidly as the effective hydraulic diameter decreases, even a modest reduction can substantially decrease the capillary-driven flow velocity.^8,18,19^

**Table 2:** Comparison with the Literature-Reported Capillary-Driven Flow in Nanofluidic Channels

	our study	Mozaffari *et al*.^14^	Oh *et al*.^13^
Channel wall material and size	Si_3_N_4_ and GaN 200 nm bottom side length (of a triangle channel)	Borofloat glass 15 μm width and 47 nm height (of a rectangular channel)	SiOx and silver 10 μm width (of a rectangular channel)
Fabrication process	Nanoimprint lithography	Reactive ion etching	Micromachining
Test liquid	pure methanol, pure ethanol, methanol–ethanol mixture, phosphate buffered saline and ethylenediaminetetraacetic acid	bitumen, water, pure ethanol, pure methanol and bitumen	water, pure methanol, pure ethanol and methanol–ethanol mixture
Velocity	1–15 μm/s (average velocity)	water: 700 μm/s, pure methanol: 600 μm/s, pure ethanol: 200 μm/s, 20% bitumen: 130 μm/s, 40% bitumen: 40 μm/s (penetration distance: 0.5 mm)	185–382 μm/s (average velocity)

### 3.3 Nanofluidic Channel Morphology

Inspired by the efficient fluid transport mechanisms observed in plant vascular systems, this study developed a biomimetic nanofluidic platform. The arrangement and cross-sectional geometry of the fabricated nanofluidic channels were designed to mimic the structural features of vascular transport elements found in plant vascular bundles.^20^ Such biological architectures have evolved to facilitate passive fluid transport over long distances and therefore provide valuable design inspiration for capillary-driven nanofluidic systems. The nanofluidic channel structures were fabricated through a combination of nanoimprint lithography and thin-film deposition processes. During PECVD deposition, Si_3_N_4_ was formed through the reaction of silicon- and nitrogen-containing precursors. Owing to the deposition characteristics of the process, lateral film growth gradually exceeded the remaining opening width, resulting in the formation of enclosed isosceles triangular nanofluidic channels. The resulting geometry resembles vascular transport elements and provides a unique nanofluidic channel architecture for capillary-driven flow. An additional advantage of this fabrication strategy is its geometric tunability. By adjusting deposition conditions and structural parameters, the cross-sectional dimensions and nanofluidic channel geometry can be readily modified to satisfy the requirements of different applications. Furthermore, the use of optically transparent semiconductor materials, including GaN and Si_3_N_4_, facilitates direct visualization of fluid transport behavior, which is advantageous for experimental observation and characterization.

### 3.4 Numerical Evaluations

#### 3.4.1 Nanofluidic Channel Geometry and Material Configuration

We investigated a nanofluidic channel with an isosceles triangular cross section, consisting of a bottom wall length *a* and two identical side walls, each of length 6*a*. The nanofluidic channel had a total length of 80*a*. The bottom wall was composed of GaN, whereas the two side walls were composed of Si_3_N_4_, resulting in heterogeneous surface wettability. The height of the triangular cross section *h* is given by

2 $$h=\sqrt{({6a)}^{2}-{\left(a/2\right)}^{2}}=\frac{\sqrt{143}}{2}a$$

leading to a cross-sectional area *A*

3 $$A=\frac{1}{2}\mathrm{ah}=\frac{\sqrt{143}}{4}a^{2}$$

The wetted perimeter *P* is

4 P=a+6a+6a=13a

and the corresponding hydraulic diameter *D*_h_ is

5 $$D_{h}=\frac{4A}{P}=\frac{\sqrt{143}}{13}a$$

#### 3.4.2 Capillary Pressure in a Heterogeneous Triangular Nanofluidic Channel

For a polygonal channel with spatially heterogeneous wettability, the capillary pressure Δ*P*_c_ can be expressed as a perimeter-weighted Young–Laplace contribution^21^

6 $$\Delta P_{c}=\gamma \frac{\sum_{i}L_{i}\cos\theta_{i}}{A}$$

where γ, *L*_*i*_ and *θ*_*i*_ denote the surface tension, wetted length and contact angle of each wall segment. In the present geometry, this yields

7 $$\Delta P_{c}=\frac{4\gamma }{\sqrt{143}a}\left({\cos\theta }_{\mathrm{GaN}}+12{\cos\theta }_{\mathrm{Si}3N4}\right)$$

#### 3.4.3 Pressure Drop

Assuming incompressible Newtonian flow, a no-slip boundary condition, negligible gravity, and fully developed laminar flow driven solely by capillary pressure in a non-circular duct, the pressure drop is related to the average velocity *U*_avg_ by

8 $$\Delta P=\frac{2\mathrm{Po}\mu U_{\mathrm{avg}}L}{D_{h}^{2}}$$

where Po is the Poiseuille number, which depends solely on the cross-sectional shape^8,18,19^ and μ is the dynamic viscosity. The apex angle of the triangular cross section is approximately 9.56°. For the triangular duct with the apex angle near 10°, the Poiseuille number is approximated as 12.47.

#### 3.4.4 Average Wall Shear Stress

The perimeter-averaged wall shear stress for fully developed laminar flow can be obtained from a force balance between pressure and viscous shear,^8,18,19^

9 $$\tau_{\mathrm{avg}}=\frac{\Delta PA}{PL}$$

Using the capillary pressure expression and the geometric relations above, this reduces to

10 $$\tau_{\mathrm{avg}}=\frac{\gamma }{1040a}\left({\cos\theta }_{\mathrm{GaN}}+12{\cos\theta }_{\mathrm{Si}3N4}\right)$$

#### 3.4.5 Numerical Evaluation Results

The experimentally measured values were substituted into eqs ([Disp-formula eq2])–([Disp-formula eq10]) to calculate capillary pressure, pressure drop, and average wall shear stress. Because the low solute concentrations of 1× phosphate-buffered saline and 1 mM ethylenediaminetetraacetic acid (EDTA) were assumed to produce negligible changes in the relevant fluid properties, their surface tension and dynamic viscosity at 25 °C were approximated using those of water. The surface tension and dynamic viscosity of water are ∼72.0 mN/m (γ)^22^ and ∼0.890 mPa·s (μ), respectively.^23^ The static contact angles were experimentally measured by using a contact angle analyzer (FTA1000, First Ten Angstroms, Inc., USA). Contact angles of 1× phosphate buffered saline and 1 mM ethylenediaminetetraacetic acid on GaN and Si_3_N_4_ for the numerical evaluations were experimentally measured as 75.56°, 57.88°, 31.06° and 36.38°, respectively (Supporting Information, Video S4). The fabricated nanofluidic channels have a bottom width of approximately 200 nm (*a* = 200 nm). The surface tension and dynamic viscosity of pure methanol and pure ethanol are approximately ∼22.17 mN/m (γ),^24^ ∼0.544 mPa·s (μ),^24^ ∼21.91 mN/m (γ)^24^ and ∼1.074 mPa·s (μ).^24^ Regarding the surface tension and dynamic viscosity of methanol–ethanol mixtures, the surface tension of methanol–ethanol mixtures was estimated using a linear mole-fraction mixing rule, whereas the dynamic viscosity was estimated using the Arrhenius logarithmic mixing rule:^25,26^

11 $$\gamma_{\mathrm{mix}}\approx x_{\mathrm{MeOH}}\gamma_{\mathrm{MeOH}}+x_{\mathrm{EtOH}}\gamma_{\mathrm{EtOH}}$$

12 $$\ln\mu_{\mathrm{mix}}\approx x_{\mathrm{MeOH}}{\ln\mu }_{\mathrm{MeOH}}+x_{\mathrm{EtOH}}{\ln\mu }_{\mathrm{EtOH}}$$

The calculated surface tension and dynamic viscosity of methanol–ethanol mixtures are listed in Table 3.

**Table 3:** Surface Tension and Dynamic Viscosity of Pure Methanol, Methanol–Ethanol Mixtures and Pure Ethanol

test liquid	surface tension (mN/m)	dynamic viscosity (mPa·s)
100% methanol	22.17	0.544
80% methanol + 20% ethanol	22.13	0.607
60% methanol + 40% ethanol	22.09	0.696
40% methanol + 60% ethanol	22.04	0.798
100% ethanol	21.91	1.074

We also measured the static contact angles of pure methanol, pure ethanol, and methanol–ethanol mixtures on GaN and Si_3_N_4_ (Table 4 and Supporting Information, Video S4).

**Table 4:**
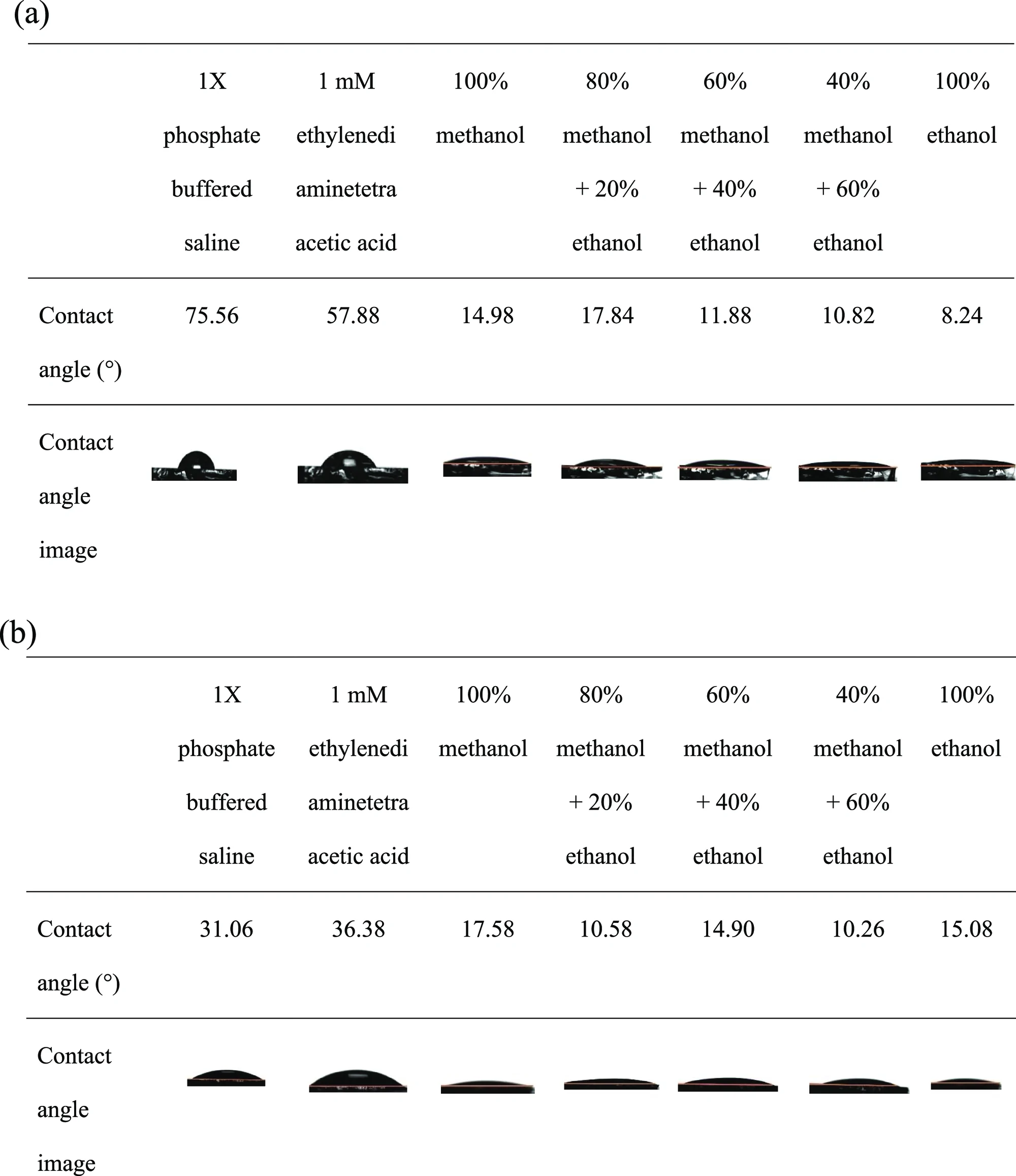
Measured Contact Angles of (a) GaN and (b) Si_3_N_4_ with Different Test Liquids

Substituting these parameters yields the numerical evaluation results regarding the calculated capillary pressure, pressure drop and average wall shear stress, as listed in Table 5. The numerical evaluations of capillary pressure, pressure drop, and average wall shear stress were performed based on the experimentally measured average flow velocity. Because this required input parameter was unavailable for pure ethanol, these quantities could not be calculated and were therefore not included in Table 5. The numerical framework further indicates that replacing Si_3_N_4_ with another semiconductor material having different wettability characteristics could alter the capillary pressure. Fabrication-induced rounding of the triangular corners or partial channel obstruction may effectively reduce the hydraulic diameter of the nanofluidic channel. Because hydraulic resistance exhibits a strong inverse power-law dependence on the characteristic channel dimension,^8^ even a modest reduction in the effective hydraulic diameter (on the order of several tens of nanometers) can substantially increase flow resistance. Consequently, the capillary-driven flow velocity may decrease by an order of magnitude, leading to corresponding changes in capillary pressure, pressure drop, and average wall shear stress.

**Table 5:** Calculated Capillary Pressure, Pressure Drop, and Average Wall Shear Stress with Different Test Liquids^a^

test liquid	capillary pressure (MPa)	pressure drop (Pa)	average wall shear stress (kPa)
100% methanol	0.46	47	1.32
80% methanol + 20% ethanol	0.471	27	1.36
60% methanol + 40% ethanol	0.464	95	1.34
40% methanol + 60% ethanol	0.471	68	1.36
1× phosphate buffered saline	1.27	39	3.65
1 mM ethylenediaminetetraacetic acid	1.23	113	3.52

a Note: The capillary pressure, pressure drop, and average wall shear stress were calculated using the experimentally measured average flow velocity. Because pure ethanol did not propagate through the single-layer nanofluidic channels, these parameters could not be calculated and are therefore not included in this table.

## 4 Conclusion

Heterogeneous GaN and Si_3_N_4_ nanofluidic channels with single-layer and triple-layer architectures were successfully fabricated using nanoimprint lithography and thin-film deposition processes. Using the proposed fabrication process, single-layer nanofluidic channels with a bottom width of approximately 200 nm were successfully fabricated. Triple-layer nanofluidic channel structures were also successfully constructed, demonstrating the feasibility of extending the proposed fabrication strategy to complex multilayer architectures. Experimental visualization demonstrated the feasibility of capillary-driven flow transport inside the fabricated triangular nanofluidic channels, while analytical evaluations of single-layer nanofluidic channels provided quantitative insights into capillary pressure, pressure drop, and average wall shear stress. The measured flow velocities inside single-layer nanofluidic channels for the test liquids ranged from 0.17 to 16.86 μm/s. Based on the experimentally measured flow velocities, the calculated capillary pressures, pressure drops, and average wall shear stresses were 0.46–1.27 MPa, 27–113 Pa, and 1.32–3.65 kPa, respectively. The measured flow velocities were generally one to two orders of magnitude lower than the values reported in selected previous studies, likely reflecting differences in channel geometry, dimensions, surface properties, and experimental conditions. Successful fabrication of multilayer nanofluidic channel structures demonstrated the capability of nanoimprint lithography-based processes for constructing hierarchical semiconductor nanofluidic architectures. This work provides a useful framework for understanding capillary-driven flow transport in nanofluidic channels and offers practical design guidelines for future nanofluidic devices used in biomedical and analytical applications.

## Supplementary Material











## Data Availability

The data and materials for this study are available from the corresponding authors upon reasonable request.

## Notes

Experimental setup and uncertainty analysis (PDF)
Methanol–ethanol mixture flows in single-layer nanofluidic channels (Video S1) (MP4)
1× Phosphate buffered saline and 1 mM ethylenediaminetetraacetic acid flows in single-layer nanofluidic channels (Video S2) (MP4)
Pure methanol and pure ethanol flows in triple-layer nanofluidic channels (Video S3) (MP4)
Static contact angles on GaN and Si_3_N_4_ (Video S4) (MP4)
