# Supplementary material for: Capillary-Driven Flow in Triangular Nanofluidic Channel Fabricated via Nanoimprint Lithography with Heterogeneous Semiconductor Materials
Source: ACS Omega. 2026 Sep 15;11(38):58091–9. doi: 10.1021/acsomega.6c07925 (PMC13629386; doi:10.1021/acsomega.6c07925)
Supplement: Supplementary file 1 [file ao6c07925_si_001.pdf]

Capillary-Driven Flow in Triangular Nanofluidic Channel Fabricated via Nanoimprint Lithography with Heterogeneous Semiconductor Materials

Yi-Hsin Wang[1], Kang-Yuan Lee[2], Chung-Hsiang Lin[2*] and Chao-Min Cheng[1*]

[1]Institute of Biomedical Engineering, National Tsing Hua University, Hsinchu, Taiwan

[2]Quantum NIL Corporation, Miaoli, Taiwan

E-mail: chaomin@mx.nthu.edu.tw & slin@qtnil.com (corresponding authors)

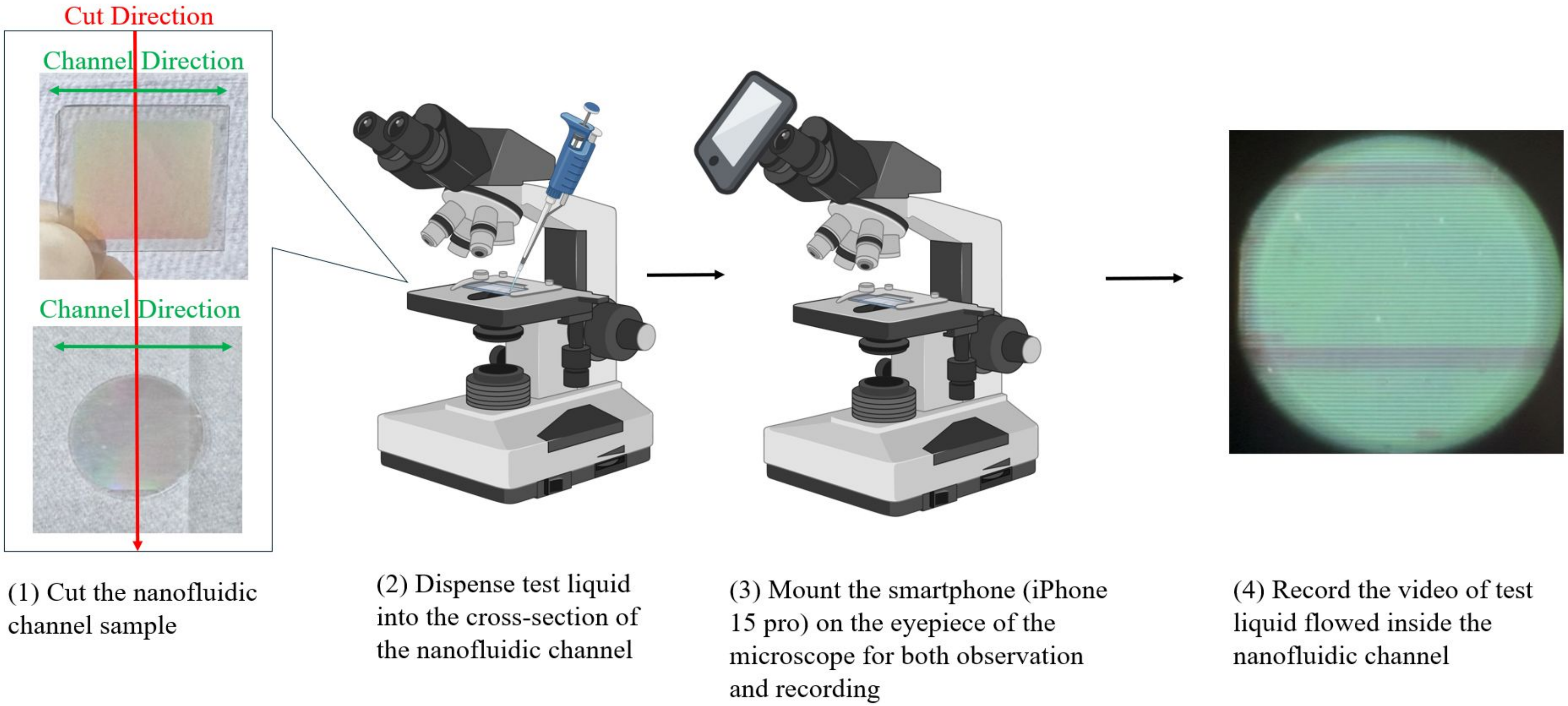

Figure S1. Experimental setup of the capillary-driven flow (inside nanofluidic channels) observations.

Uncertainty analysis

(1) Temporal uncertainty

The videos were recorded at 24 frames s^{-1}, corresponding to a frame interval of

$$\Delta t = \frac{1}{24} = 0.0417\ s$$

The liquid front was manually identified from the recorded frames. Assuming an uncertainty of one frame for both the initial and final positions, the combined temporal uncertainty was estimated as

$$u_t = \sqrt{\left(\frac{1}{24}\right)^2 + \left(\frac{1}{24}\right)^2} = 0.059\ \text{s}$$

(2) Spatial uncertainty

The liquid-front displacement was measured directly from the video images using a ruler with a minimum division of 1 mm. Assuming a reading uncertainty of one-half of the minimum division, the uncertainty associated with a single position measurement was 0.5 mm. Since the displacement was obtained from the difference between the initial and final positions, the combined spatial uncertainty was

$$u_z = \sqrt{(0.05)^2 + (0.05)^2} = 0.071\ \text{cm}$$

The same uncertainty was assigned to the measured image length

$$u_x = 0.071\ \text{cm}$$
